# Targeting cognitive biases to improve social cognition and social emotional health

**DOI:** 10.3389/fpsyg.2025.1534125

**Published:** 2025-03-19

**Authors:** Susan A. J. Birch, Charlotte I. Stewardson, Kate Rho, Ashali Kataria, Shannon M. Craig, Minh D. H. Phan, Irene Savi, Kseniia Voronkova, Jenny Lee, Gayatri Choudhary, Diba Torjani

**Affiliations:** Department of Psychology, University of British Columbia, Vancouver, BC, Canada

**Keywords:** social cognition, cognitive bias, theory of mind, social emotional health, cognitive debiasing, intervention, education, development

## 1 Introduction

Social cognitive skills are crucial for understanding and navigating human interactions, enabling us to process, interpret, and respond to social information (Arioli et al., 2018). A key component of these skills is *theory of mind*, which involves inferring and reasoning about one's own and others' mental states, including beliefs, intentions, desires, thoughts, and emotions (Premack and Woodruff, 1978; Wimmer and Perner, 1983). Theory of mind is essential in almost every social interaction, as it helps us understand human actions (e.g., Baron-Cohen, 1995; Frith and Frith, 2005), underpins cultural learning (e.g., Henrich, 2004; Herrmann et al., 2007), and is vital for effective communication and social decision-making (e.g., Baron-Cohen, 1995; Birch et al., 2017; Haddock and Birch, 2024). Theory of mind has also been shown to promote prosocial behavior (e.g., Imuta et al., 2016) and reduce prejudice (Shih et al., 2009).

Philosopher Elbert G. Hubbard aptly stated, “If men [sic] could only know each other, they would never idolize nor hate” (Hubbard, 1911, p. 13). We interpret this to mean that a rich understanding of each other's perspectives fosters greater social harmony and social emotional health. More specifically, we believe that by using *theory of mind* to understand others' perspectives, people can recognize their shared humanity and overcome the tendencies to either idealize or condemn others. This understanding may also help reduce biases and assumptions that lead to flawed social judgments, such as the “black-and-white” thinking in which others are seen as either flawless or completely flawed.

Consistent with Hubbard's sentiments, we propose that interventions enhancing social cognitive skills can significantly improve social-emotional health. Furthermore, we predict that the most successful interventions will incorporate strategies to minimize cognitive biases—systematic errors in thinking that affect decision-making and behavior (Tversky and Kahneman, 1974; for a review, see Ellis, 2018). We support this view by briefly reviewing research that shows: (a) enhancing social cognition improves various aspects of social-emotional health, (b) cognitive biases play a critical role in the link between social cognition and social-emotional health, and (c) strategies for reducing cognitive biases have tremendous promise for enhancing social cognition and social emotional health.

## 2 Improving social cognition can improve social-emotional health

Research has consistently demonstrated that theory of mind abilities are pivotal for developing and maintaining social relationships, particularly during childhood (Dunn and Cutting, 1999; Peterson et al., 2016; Etel and Slaughter, 2019; for reviews see Repacholi and Slaughter, 2003; Haddock and Birch, 2024). Evidence on the relationship between theory of mind and social competencies in adults, however, has been somewhat more mixed (Bora and Berk, 2016; Davis, 1983; Livingston et al., 2024; Wolgast et al., 2020). Generally speaking, individual differences in theory of mind are present early and continue into adulthood, with more advanced theory of mind predicting several positive outcomes (Dunn and Cutting, 1999; Repacholi and Slaughter, 2003). For example, higher theory of mind scores are associated with greater social understanding, higher levels of empathy, and more prosocial behavior, leading to reduced interpersonal conflicts and increased relationship satisfaction (e.g., Davis, 1983; Repacholi and Slaughter, 2003; for two meta-analyses see Imuta et al., 2016; Slaughter et al., 2015). Similarly, more advanced theory of mind has been associated with increased cooperation (Etel and Slaughter, 2019) as well as increased communication and reduced peer conflict (Dunn and Cutting, 1999; Haddock and Birch, 2024). Studies by Peterson et al. (2015) demonstrated that higher theory of mind is associated with increased self-esteem and higher quality friendships. Peterson et al. (2016) also found that children's theory of mind understanding independently predicted social skills above and beyond age, gender, and verbal ability. Furthermore, more advanced theory of mind has also been shown to reduce the risk of social adversity, such as bullying and social exclusion (Bosacki et al., 2020; Smith, 2017). More advanced theory of mind also appears to act as a protective factor against trauma and adversity (e.g., Cadamuro et al., 2016; Hughes and Ensor, 2006, 2007). Conversely, poor theory of mind skills are associated with greater psychological distress (Wolgast et al., 2020), more emotional symptoms, and increased loneliness (Caputi and Schoenborn, 2018). This latter result is especially noteworthy given longitudinal studies linking loneliness to a variety of negative health outcomes, including poorer sleep quality (Cacioppo et al., 2002), and increased depressive symptoms (Cacioppo et al., 2010). Even in adulthood, theory of mind predicts emotional symptoms such as sadness and depression. A meta-analysis of 18 studies examining the relationship between theory of mind and Major Depressive Disorder in adults revealed that deficits in theory of mind can be a risk factor for depression and accompanying psychosocial impairment, with the level of theory of mind impairment predicting symptom severity (Bora and Berk, 2016).

## 3 Maximize social cognition by minimizing cognitive biases

Decades of research across the psychological sciences have shown that cognitive biases play a critical role in shaping our perceptions, decisions, and interactions, influencing nearly every aspect of human interaction (Tversky and Kahneman, 1974; Kahneman, 2011; for a review, see Ellis, 2018). These cognitive biases are normal by-products of how the mind works; nonetheless, individual differences in the magnitude of these biases predict a range of outcomes. Cognitive biases lead to errors in decision-making and social judgments, impede communication, contribute to maladaptive behaviors, and even play a role in mental health conditions like depression (Beck, 1979; Kahneman, 2011; Nisbett and Ross, 1980; Tversky and Kahneman, 1974).

Not surprisingly, the way we think *about others* and their mental states is also vulnerable to cognitive biases. Given their social elements, cognitive biases are sometimes referred to as social cognitive biases. Social cognitive biases, systematic tendencies, or errors, in the way we think about others and their mental states, can be particularly damaging to interpersonal relationships, impair communication, and lead to poor social decision-making (e.g., Birch and Bernstein, 2007; Nickerson, 1999; Savitsky et al., 2011). For instance, consider the spotlight effect which occurs when individuals overestimate the extent to which others notice and evaluate their actions and appearance (Gilovich et al., 2000). This can lead to heightened self-consciousness and increased social anxiety, as individuals mistakenly believe they are under scrutiny. For example, in contexts like volleyball games and video games, participants overestimated how much their teammates notice differences in their performance compared to a typical game and anticipated harsher evaluations than were actually given (Gilovich et al., 2000, 2002). This tendency for individuals to feel that they are the center of attention, especially in potentially unfavorable situations, is linked to increased self-consciousness and social anxiety (e.g., Brown and Stopa, 2007).

Another cognitive bias that plays a clear role in social cognition is the curse of knowledge bias. The curse of knowledge bias refers to the tendency to be swayed by one's knowledge when attempting to reason about a more naive perspective (e.g., Birch and Bloom, 2003; Bernstein et al., 2004; Camerer et al., 1989; Fischhoff, 1977; Taylor et al., 1994; Sutherland and Cimpian, 2015; for a meta-analyses of 122 studies see Christensen-Szalanski and Willham, 1991). A classic example of the curse of knowledge bias (sometimes called ‘hindsight bias') is when adults who know the outcome of an event (e.g., a sports game, an election, or a battle) overestimate how likely others are to predict that outcome. In contrast, adults who do not know the event's outcome tend to make more accurate estimates of what others will predict (e.g., Blank et al., 2003; Fischhoff, 1975; Fischhoff and Beyth, 1975, for review see Ghrear et al., 2016; for a meta-analyses see Guilbault et al., 2004). Given that the curse of knowledge bias leads individuals to overestimate how common their knowledge is, it regularly impacts communication and social judgments in various ways (e.g., Birch, 2005; Camerer et al., 1989). For example, experts often assume that their specialized knowledge is more widely understood than it is, which can hinder effective communication and lead to misunderstandings (Hinds, 1999). Importantly, research has shown that one of the most widely-used tasks to measure theory of mind, the classic ‘false belief task,' is heavily influenced by the curse of knowledge bias. Although the curse of knowledge and false belief reasoning appear to be independent constructs with different developmental patterns (Bernstein, 2021), experimentally reducing this bias has been shown to improve false belief reasoning in both children (Ghrear et al., 2021) and adults (Birch and Bloom, 2007; see also Ghrear et al., 2020; Keysar et al., 2003). Importantly, interventions that provide contextual feedback about others' perspectives appear particularly effective at minimizing this type of ‘egocentric bias' in adults (Damen et al., 2021).

Another well-documented cognitive bias in social cognition is the hostile attribution bias, which refers to the tendency to interpret ambiguous or neutral social cues as being intentionally hostile or aggressive (Dodge and Crick, 1990). The hostile attribution bias can significantly affect how individuals perceive and react to social interactions, because it shapes how people interpret the intentions of others. For instance, someone prone to this bias may interpret an accidental bump in a crowded hallway as a deliberate act of aggression and react aggressively in response. This bias overlaps with a broader phenomenon known as ‘interpretation bias,' a widely studied bias in clinical research. Interpretation bias is a type of negativity bias involving the tendency to interpret ambiguous or neutral information in a negative manner. This bias is associated with higher levels of stress, anxiety, and depression (e.g., Mathews and MacLeod, 2002). In fact, research shows this cognitive bias is not merely associated with mood disorders but actively contributes to their development and maintenance (Gotlib and Joormann, 2010; Mathews and Mackintosh, 2000; Mathews and MacLeod, 2002; Kindt and Van Den Hout, 2001). Interpretation biases may also be associated with psychotic symptoms. That is, negative interpretation bias such as hostile attribution bias tends to be more pronounced among individuals who are experiencing both clinical and subclinical levels of psychosis, though the quality of some of these studies varies (for a review, see Samson et al., 2024; see also Beck and Clark, 1997). Interestingly, it has been theorized that negative interpretation biases might explain the increased rates of social withdrawal among individuals with subclinical and clinical levels of psychotic symptoms (e.g., negative interpretations of social interactions could reinforce a tendency to isolate; Rector and Beck, 2002). This latter observation reinforces the point that minimizing social cognitive biases have tremendous potential for improving social emotional health. The aforementioned biases are only a few examples of cognitive biases that affect social cognition. There are many others; several of which may share underlying mechanisms (e.g., Birch and Bloom, 2003; Birch and Bernstein, 2007; Oeberst and Imhoff, 2023; Tversky and Kahneman, 1974).

Fortunately, the ability to minimize many cognitive biases has been well-documented (e.g., Ross et al., 1977; Hirsch et al., 2018; Hooper et al., 2015; Macrae et al., 2016). For example, training to reduce negativity biases, such as interpretation bias, in individuals with a history of depression, has been shown to lessen the severity of depressive symptoms (Hirsch et al., 2018; Hofmann et al., 2012). Similarly, cognitive debiasing interventions with individuals with hostile attribution bias have also been shown to be effective (e.g., Hiemstra et al., 2018). For individuals with schizophrenia spectrum disorders, metacognitive training (MCT), an evidence-based intervention addressing cognitive biases over 8 to 16 sessions, has been shown to effectively improve global social cognition and theory of mind, with adapted versions being used with other clinical populations such as individuals with major depressive disorder, obsessive-compulsive disorder and borderline personality disorder (for a review, see Hotte-Meunier et al., 2024). While some debiasing techniques involve lengthy and/or implicit debiasing techniques, approaches that educate individuals about cognitive biases and/or offer strategies to lessen them can also be highly effective (e.g., Morewedge et al., 2015; Gilovich et al., 2000; van Brussel et al., 2021). For instance, even a brief 30–60 min intervention educating individuals about biases and ways to address them resulted in significant bias reductions for at least 2 to 3 months (Morewedge et al., 2015). Similar research suggests that game-based formats and spaced reminders may be especially beneficial for minimizing bias (Clegg et al., 2014). These latter examples did not specifically examine the broader benefits for social cognition, nonetheless, we believe these types of debiasing strategies hold great promise for enhancing social cognition and several facets of social-emotional health (see Craig et al., 2024 for a recent review).

## 4 Conclusion

Reasoning about the minds of others is multifaceted—it is complex and nuanced. A recent review of theory of mind measures suggested that there are at least 39 different theory of mind sub-abilities (Beaudoin et al., 2020). Just as researchers should avoid relying on a single measure of theory of mind (e.g., Bloom and German, 2000; Haddock and Birch, 2024), we should also refrain from depending on any single intervention approach. Vast individual differences exist in people's strengths and limitations in reasoning about the minds of others. As such, we believe combining multiple strategies is the best way to address the multifaceted nature of theory of mind and the unique and diverse challenges individuals face.

Notably, some cognitive biases appear to play an even greater role during childhood and early adolescence than in adulthood (e.g., Birch, 2005; Bernstein et al., 2011; Ghrear et al., 2021, 2020), highlighting the importance of introducing debiasing strategies in younger populations. Educating parents and teachers about cognitive debiasing strategies also has considerable merit and can provide valuable indirect benefits in situations where directly teaching strategies to very young children might be challenging. For instance, Gehlbach and Vriesema (2019) suggest that educating individuals about cognitive biases and related theories equips them with tools to identify and create learning opportunities for children. These opportunities encourage children to reassess their perspectives during social interactions and conflicts, ultimately helping them reduce their biases. Addressing these biases early in development has the greatest potential to prevent social-emotional problems and yield the most long-term benefits—for individuals and for society as a whole.

In conclusion, it is our opinion that intervention approaches can maximize social cognition by minimizing cognitive biases. To be clear, we are not advocating a cognitive debiasing approach should replace existing intervention techniques, but rather that cognitive debiasing strategies be *integrated* with existing approaches. We believe that the most effective interventions for enhancing social cognition and social emotional health will combine existing methods with education on cognitive biases and concrete strategies to overcome them.
